# Supplementary material for: Causal Relationship between Aging and Anorexia Nervosa: A White-Matter-Microstructure-Mediated Mendelian Randomization Analysis
Source: Biomedicines. 2024 Aug 16;12(8):1874. doi: 10.3390/biomedicines12081874 (PMC11351342; doi:10.3390/biomedicines12081874)
Supplement: Supplementary file 1 [file biomedicines-12-01874-s001.zip › Supplementary Figure/Figure S4/Singe SNP plot of MR analysis for TL to ubm-b-2010.pdf]

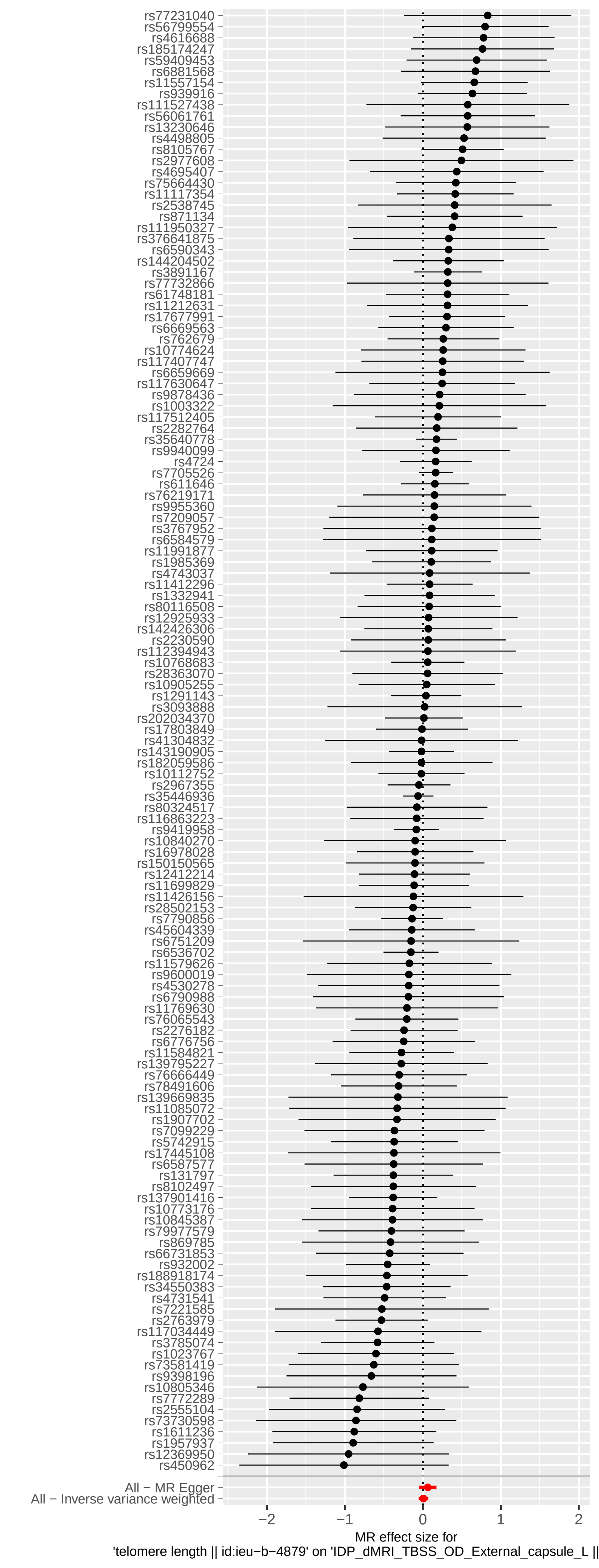
rs77231040
rs56799554
rs4616688
rs185174247
rs59409453
rs6881568
rs11557154
rs939916
rs111527438
rs56061761
rs13230646
rs4498805
rs8105767
rs2977608
rs4695407
rs75664430
rs11117354
rs2538745
rs871134
rs111950327
rs376641875
rs6590343
rs144204502
rs3891167
rs77732866
rs61748181
rs11212631
rs17677991
rs6669563
rs762679
rs10774624
rs117407747
rs6659669
rs117630647
rs9878436
rs1003322
rs117512405
rs2282764
rs35640778
rs9940099
rs4724
rs7705526
rs611646
rs76219171
rs9955360
rs7209057
rs3767952
rs6584579
rs11991877
rs1985369
rs4743037
rs11412296
rs1332941
rs80116508
rs12925933
rs142426306
rs2230590
rs112394943
rs10768683
rs28363070
rs10905255
rs1291143
rs3093888
rs202034370
rs17803849
rs41304832
rs143190905
rs182059586
rs10112752
rs2967355
rs35446936
rs80324517
rs116863223
rs9419958
rs10840270
rs16978028
rs150150565
rs12412214
rs11699829
rs11426156
rs28502153
rs7790856
rs45604339
rs6751209
rs6536702
rs11579626
rs9600019
rs4530278
rs6790988
rs11769630
rs76065543
rs2276182
rs6776756
rs11584821
rs139795227
rs76666449
rs78491606
rs139669835
rs11085072
rs1907702
rs7099229
rs5742915
rs17445108
rs6587577
rs131797
rs8102497
rs137901416
rs10773176
rs10845387
rs79977579
rs869785
rs66731853
rs932002
rs188918174
rs34550383
rs4731541
rs7221585
rs2763979
rs117034449
rs3785074
rs1023767
rs73581419
rs9398196
rs10805346
rs7772289
rs2555104
rs73730598
rs1611236
rs1957937
rs12369950
rs450962
All − MR Egger
All − Inverse variance weighted
−2
−1
0
1
2
MR effect size for
'telomere length || id:ieu−b−4879' on 'IDP_dMRI_TBSS_OD_External_capsule_L ||
